# The Future of Motion Preservation and Arthroplasty in the Degenerative Lumbar Spine

**DOI:** 10.3390/jcm14103337

**Published:** 2025-05-11

**Authors:** Michael S. Pheasant, Matthew W. Parry, Mina Girgis, Alex Tang, Tan Chen

**Affiliations:** 1 Geisinger Musculoskeletal Institute, Danville, PA 17822, USA; 2 Division of Orthopedic Spine Surgery, Geisinger Musculoskeletal Institute, Danville, PA 17822, USA

**Keywords:** lumbar spine degeneration, lumbar arthroplasty, motion-preserving surgery

## Abstract

The lumbar degenerative cascade is a pathological process that affects most of the aging adult population and has significant negative economic consequences. Lumbar fusion surgery remains a mainstay of treatment for refractory degenerative disease but carries significant long-term consequences. More recently, lumbar arthroplasty and motion-sparing technology has become an increasingly popular alternative surgical option in carefully indicated patients. Arthroplasty technology carries the theoretical benefits of spinal segment motion preservation and decreased degeneration of adjacent segments as compared to traditional fusion procedures. This article will review the lumbar degenerative cascade and its related anatomic considerations, current management strategies and the challenges surrounding lumbar spinal fusion, including adjacent segment disease. This article will also review the theoretical benefits of lumbar arthroplasty and motion preservation. Furthermore, this paper will highlight the current state of lumbar arthroplasty, including current concepts of implant design, limitations, outcomes and ongoing development. It will review the development and current state of artificial disk arthroplasty, total joint arthroplasty and posterior column motion-preserving implants, including flexible rods and facet joint replacement.

## 1. Introduction

Degeneration of the lumbar spine is a significant contributor to disability throughout the world, with an incidence of 3.63% worldwide [1]. Lumbar degeneration is a complex process which encompasses disk degeneration, lumbar spinal stenosis, facet arthropathy and deformity [2]. The degenerative process involves the complex interplay between the anterior and posterior column and presents a challenge for management as there may be numerous pain generators involved. The resultant low back pain can have a major impact on function and quality of life [3].

To date, the most common surgical strategy for lumbar degeneration is lumbar fusion, which has long been considered the gold standard [4]. Despite the segmental loss of motion that occurs with fusion, the lumbar spine, unlike the appendicular skeleton, can compensate for loss of motion with a compensatory increase in the adjacent segments, reducing the functional burden of arthrodesis in the short term and leading to an acceptable clinical result. Furthermore, rapid advances in spinal fusion technologies have in part driven the use of fusion in practice [5]. Despite its widespread use, spinal fusion has drawn scrutiny over its multiple drawbacks, including pseudoarthrosis, adjacent segment pathology, hardware failure and additional surgery. Given these factors, there has been long-standing interest in motion-preserving surgery [6,7].

The concept of motion preservation in lumbar spine surgery has made several technological and clinical advances and has shown promising results in the literature, but widespread adoption remains limited. This has been due to several factors, mainly concerns over the longevity of arthroplasty implants and potential need for revision. Furthermore, the triad of joints that make up a single spinal level complicates the prospect of resurfacing and motion preservation from an implant development standpoint and from the perspective of patient selection. For example, concomitant facet arthropathy in the setting of disk degeneration would contraindicate isolated disk arthroplasty which would not address the degeneration of the posterior column. These considerations make patient selection and surgical indications challenging. Despite these hurdles, there have been several significant advances and milestones reached in lumbar arthroplasty, which will likely accelerate the use of arthroplasty implants in the coming years [5]. This article will provide a narrative review of the historical and current concepts along with the future direction of lumbar arthroplasty.

## 2. Materials and Methods

In this narrative review, the historical background, current concepts and future direction of lumbar arthroplasty will be described and discussed. To this end, current data, along with case reports and articles of historical significance, will be utilized to illustrate the historical context, current concepts and clinical experience thus far.

This article will discuss the current concepts of the lumbar degenerative cascade and the anatomical effects on the disc, ligaments and facet joints; the theoretical benefits of lumbar arthroplasty and motion preservation; and the disadvantages of spinal fusion.

Furthermore, the review will address specific implant categories, including artificial lumbar disc arthroplasty, facet joint replacement, dynamic stabilization and total joint arthroplasty. We will discuss implant designs, along with the respective advantages and disadvantages of each.

## 3. Current Concepts on the Lumbar Degenerative Cascade and the Anatomical Effects on the Disc, Ligaments and Facet Joints

Spine degeneration is a complex process which occurs with aging but can also occur independently in the setting of early degeneration [2]. The lumbar spine is a three-joint complex with intervertebral discs and two facet joints which biomechanically collaborate to distribute physiologic loads and stresses [8]. In the lumbar spine, the facet joints are designed to prevent rotational instability while providing flexibility in the sagittal plane. The interplay between the anterior and posterior columns is complex and has been well studied throughout the past decades.

The degenerative cascade was described by Kirkaldy-Willis in the 1970s and predicably follows a three-phase process. In phase one, trauma in the form of torsional stress causes damage to the anulus fibrosus, which weakens the circumferential structure of the annulus. This leads to instability in the disk in phase two, at which point the nucleus pulposus can migrate, leading to a loss of disk height, or the nucleus migrates through the anulus, which occurs in the setting of a herniation. In either case, the compensatory re-stabilization process leads to increased bony hypertrophy in the form of osteophytosis [9].

In comparison, during normal aging, disk degeneration can occur following a three-phase molecular pattern. In phase one, there is an accumulation of molecular damage to protein and DNA structures the intervertebral disk over time. Subsequently, in phase two, there is a resultant change in the ECM of the disk, which in phase three, leads to a loss of biologic structure and function of the disk, with compensatory bone changes and increased stresses placed on the facet joints. The compensatory changes that then occur in the facet joints include facet degeneration and arthropathy [2].

More recently, the interplay between the intervertebral disk and facet joints has been better elucidated. Studies involving MRI review of the lumbar spine over time have demonstrated the interplay between disc degeneration and facet tropism. In many cases, disk degeneration appears to precede facet degeneration, with facet arthropathy occurring as a result of microinstability at the intervertebral disk [8]. Instability at the intervertebral disk and the subsequent imbalance in kinematic forces places increased strain on the facet joints. The facet joint undergoes synovitis, cartilage destruction, capsular laxity and, ultimately, instability. This cascade of changes leads to degenerative rotational and translational deformities, such as degenerative spondylolisthesis and degenerative scoliosis [9,10]. In other cases, a posterior-to-anterior cascade of degeneration has been observed. MRI studies have shown that variations of facet morphology appear to correlate with early disk degeneration and concomitant facet degeneration, suggesting that variations in facet morphology may alter the stress distribution on the intervertebral disk and play a role in accelerating disk degeneration [8]. Regardless of the degenerative pathway, lumbar degeneration may involve either the anterior column, the posterior elements or both, and treatment strategies need to be tailored to best address the pathology involved.

## 4. Disadvantages of Spinal Fusion

In the orthopedic treatment of degenerative joints of the appendicular skeleton, arthrodesis has been largely abandoned in favor of arthroplasty. Management of spine degeneration has been an exception, with fusion of the diseased spinal level remaining the gold standard for surgical management. Fusion of the lumbar spine is accepted as a reasonable treatment of spine degeneration, since adjacent levels can compensate for the fused level, reducing the impact of arthrodesis on functional motion and resulting in a clinically acceptable outcome [11]. Despite being the standard of care, lumbar spinal fusion has several well-established complications, including pseudoarthrosis, hardware failure and adjacent level degeneration [3,12]. These post-operative complications lead to poor and often dreaded outcomes that commonly lead to a need for revision surgery.

Adjacent segment degeneration occurs in response to the loss of motion above and/or below a fused level. The increased stresses placed on the adjacent levels in compensation for loss of motion at the fused level lead to accelerated degeneration at the adjacent levels. This is a significant concern following lumbar fusion and is a common cause of revision surgery [11]. Adjacent segment degeneration (ASDe) is defined as the radiographic changes that occur at the levels adjacent to a fused level, including disk height collapse of >20% and disk wedging > 5 degrees [13]. Adjacent segment disease (ASDi), by comparison, is defined as radiographic degeneration with concomitant symptomatic worsening. ASDi has been defined as the radiographic findings of ASDe in the setting of an Oswestry disability index score with a >20 increase from baseline and VAS score > 5 for back or leg pain at follow-up [14].

Pseudoarthrosis, another feared complication of spinal fusion, occurs when bony fusion fails to occur and pseudoarthrosis forms. Incidence of pseudoarthrosis has decreased over time as fusion techniques have improved; however, this remains a common complication of lumbar fusion with an incidence of 5–15% [15]. Similarly, hardware failure rates have also decreased over time with advances in implant technology but also remain a risk following lumbar fusion. Irmola et al. found a revision rate of 12.5% in a cohort of 433 consecutive lumbar fusion patients [16].

Given the significant complications which plague spinal fusion, it is not surprising that clinical success at 5 years is only 51% [3]. Concerns regarding these outcomes have driven an increased interest in motion-preserving treatments which has parallelled the rapid development in fusion technologies and surgical techniques.

## 5. Theoretical Benefits of Lumbar Arthroplasty and Motion Preservation

Many of the complications of spinal fusion center around the loss of motion at the fused level, adding to the appeal of motion-sparing surgery. Treatment of the degenerate joint while maintaining motion would, in theory, reduce the stresses placed at the adjacent levels at a minimum, and at best, restore native biomechanical strain. Furthermore, a motion-preserving construct eliminates the need to achieve bony fusion and the concern of pseudoarthrosis. In practice, these once-theoretical goals have been demonstrated in practice over the past decades as arthroplasty technologies have advanced; however, the literature has been limited thus far.

Successful motion preservation with lumbar arthroplasty as a proof of concept was demonstrated in several studies [11,17]. In a prospective cohort study by Rasouli et al. [17], 159 patients underwent adjacent two-level (n = 114), three-level (n = 41) or four-level (n = 4) lumbar total disk replacement (TDR). Patients were followed, and VAS-S, VAS-P, Oswestry disability index and sagittal motion on pre- and post-op radiographs at both the operative segments and adjacent segments were recorded. Patients were followed at 6 weeks, 3 months and annually from 2 to 6 years. At pre-operative radiograph, across motion segments for both groups, the mean ROM was 10.15 ± 2.71 degrees pre-operatively versus 12.30 ± 2.25 degrees post-operatively. Motion was improved at all segments except for L5-S1, where the mean pre-operative motion was 7.60 ± 3.90 versus 5.81 ± 3.1 post-operatively. Importantly, in the segment adjacent to the TDR, the mean pre-operative ROM was 8.20 ± 2.88 degrees compared with the ROM at the latest follow-up, which was 8.40 ± 2.4 degrees. Thus, there was no loss of motion demonstrated in the motion of adjacent segments in TDR patients at the 6-year follow-up [17]. These findings helped to propel lumbar arthroplasty beyond proof of concept to a viable motion-preserving alternative to lumbar fusion. Despite these promising findings, the body of evidence is limited, and additional long-term radiographic and outcome studies are needed to support the concept of motion preservation and the overall long-term efficacy of lumbar arthroplasty.

## 6. Artificial Lumbar Disc Arthroplasty

Lumbar disc replacement has become increasingly popular in the past decades as an alternative to spinal fusion and has been the leading construct in motion-preserving lumbar spine surgery. Disk replacement only addresses degeneration at the intervertebral disk; therefore, patients with concomitant facet degeneration are contraindicated, as they will have continued pain and poor outcomes following an otherwise successful disk replacement. In addition to facet degeneration, adequate bone quality, (t score > −1.0) and an absence of significant deformity are all imperative in candidates for disk arthroplasty [18]. In properly indicated patients, disk arthroplasty has shown success at both short-term and long-term follow-up [11]. Despite these promising studies, there has been concern regarding the durability and safety of disk arthroplasty. Concerns largely centered around the risk of implant stability and durability and the risk associated with revision surgery. Since most implants require an anterior approach, revision could risk damage to ureter and anterior vascular structures. A retrospective review by Schwender et al. found that revision anterior lumbar spine surgery carried a three to five times increased rate of complication when compared with primary cases [19]. Therefore, the risks of revision surgery have made durability and stability paramount factors in implant development. As the field cautiously advances, there are several implants that have been widely used and have paved the way for motion-preserving lumbar spine surgery.

The lumbar disc replacement was first developed in 1982 in Germany with the Charité artificial disc. The device consisted of an ultra-high-molecular-weight polyethylene (UHMWPE) core positioned between two polished metal end plates. The endplates featured 11 teeth used to anchor the device to the native vertebral endplate [20]. The second generation of the device featured two lateral wings with five anchoring teeth. The second-generation device experienced fractures in the end plate and was ultimately abandoned for a third-generation device developed in conjunction with Waldemar Link Gmbh & Co, (Hamburg, Germany). The Charité implant was later FDA approved in the US in 2004 and gained popularity following a prospective multicenter randomized controlled trial under FDA IDE status, which examined the Charité implant against a posterior fusion cohort. In the study, 304 patients from 14 centers were randomized into an arthroplasty group or control instrumented ALIF group at a 2:1 ratio. The outcome measures, including back pain, the Oswestry disability index and SF36 health surveys, were assessed at 6 weeks and 3, 6, 12 and 24 months post-operation. At each time point, the arthroplasty group had lower levels of disability and pain, with the exception of 24 months, which resulted in equal scores for pain. The arthroplasty group had a 1 day shorter hospital stay and expressed higher satisfaction and willingness to have the same procedure again compared to the ALIF group [21]. These results propelled disk arthroplasty forward in the spine community as a reasonable alternative to fusion in the appropriate patient.

Currently, there are two disk implants which are FDA approved for use in the US, the ProDisc-L (Centinel Spine, West Chester, PA, USA) and the activeL (Aesculap/B. Braun, Center Valley, PA, USA), approved for two and one levels, respectively, with five other implants on the global market CE marks for use in Europe: the Baguera^®^ L (SpineArt, Geneva, Switzerland), M6-L Artificial disk (Orthofix, Lewisville, TX, USA) Freedom^®^ Lumbar disk (Axiomed, Malden, MA, USA), LP-ESP^®^ Lumbar disk prosthesis (Spine Innovations, Ecully, France), Orbit™ Anterior Lumbar disk (Globus Medical, Audubon, PA, USA) Aditus Lumbar Disk Prosthesis (Aditus Medical, Berlin, Germany) and Mobidisk^®^ L Lumbar disk prosthesis (HighRidge medical, Westminster, CO, USA).

**Figure 1:**
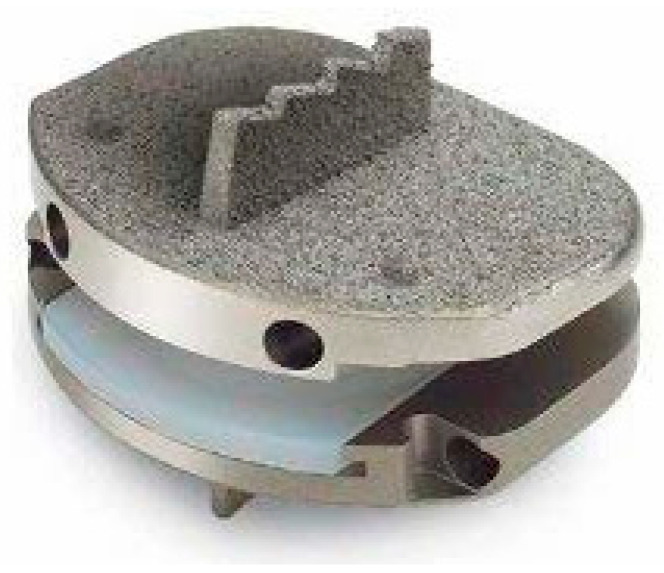
Prodisc^®^ L (courtesy of FDA SSED: https://www.accessdata.fda.gov/cdrh_docs/pdf5/P050010S020B.pdf (accessed on 24 March 2025)).

The ProDisc-L, (Centinel Spine, West Chester, PA, USA) (Figure 1) is a semi-constrained ball-and-socket design currently FDA approved for one-level or two-consecutive-level arthroplasty L3-S1. The device features a midline keel on both the superior and inferior cobalt chrome alloy endplates which house the ultra-high-molecular-weight polyethylene inlay. Both the implant footprint and lordotic angle are offered in variable sizes. The devices performed well with an FDA investigational device exemption study which compared single-level (between L3 and S1) and 360-degree spinal fusion. Single-level TDR patients treated with the ProDisc-L experienced 0% major complications, significantly higher SF36 scores, neurologic success and VAS pain scores, along with higher improvement in ODI for pain and disability scores than the fusion cohort [22]. In terms of adverse events, the device demonstrated a favorable safety profile, with persistent back pain being a leading complication but at lower rates than in their fusion counterparts. The early successful outcomes of the implant led to its FDA approval for use in two contiguous levels in 2020 [5]. The longevity of the device and lumbar disk arthroplasty at large were recently validated in a 7–21-year follow-up by Marnay et al. In this study, 1187 patients were reviewed who had undergone one-level (n = 772) and two-level (n = 415) TDRs. Notably, of the entire group, 373 patients had undergone prior surgery at the index level. All groups demonstrated marked improvements in ODI at 3 months and sustained the improvement over time, with no difference in pain scores in patients who had undergone prior surgery. Rates of revision surgery and adjacent level surgery were also low at 7 (0.67%) and 21 (1.85%) years, demonstrating the long-term clinical efficacy and durability of the ProDisk-L for TDR [11]. This study confirmed, in the long term, a low revision rate with TDR which had been seen in earlier short-term studies.

Additionally, a retrospective review of 2141 patients over a 20-year period found a 1.26% revision/removal rate. Twenty-five patients (0.99%) had implants removed (twelve patients for loosening or implant migration, three following trauma, two for ongoing pain, two for lymphocytic reaction, one for implant sizing, one for vertebral body fracture, one for implant subsidence with facet arthrosis, one for a lytic lesion and one for infection); three patients (0.12%) underwent revision to a second arthroplasty (one for core repositioning, one for core replacement due to wear and one for implant repositioning). A subset of 258 patients with a minimum 15-year follow-up were surveyed and only 1 patient underwent revision/removal after 15 years post-implant [23]. Overall, the study reiterated the longevity of these implants despite initial concerns regarding reoperation. A 2024 meta-analysis reviewing complications and clinical outcomes in lumbar disk replacement and interbody fusion was also promising in terms of reoperation risk and overall noninferiority to fusion. The 2024 retrospective study involving 1720 patients revealed no difference in EBL, length of stay, OR time, ODI leg pain, complications and reoperations between total disk replacement and lumbar fusion groups. However, the total disk replacement group had lower back pain scores [3].

Currently, in the US, FDA approval has been limited to two-level TDR. However, there has been some conflicting evidence on two-level TDR success. A 2007 study placed 99 patients into 3 groups, one-level TDR at L4-5, (n = 22), one-level TDR at L5-S1 (n = 57) and two-level TDR at L4-5 and L5-S1 (n = 20), and followed them to 24 months. The two-level fusion group had a significantly higher complication rate than the single-level group. Also of note is that this study confirmed, via fluoroscopic guided spine infiltrations, that the incidence of post-operative back pain from posterior joint structures was 9.1% (n = 2) for L4-5 TDR, 28.1% (n = 16) for L5-S1 TDR and 60.0% (n = 12) for two-level TDR (L4-5 + L5-S1) [24]. Findings such as these demonstrate a possible limitation of expanding TDR to multiple levels and also serve as a reminder of the innate inability of isolated TDR to address the posterior column. Despite these findings, recent multi-level studies have not redemonstrated a difference in one- vs multi-level TDR. In the above-mentioned trial by Rasouli et al., multi-level TDR patients uniformly demonstrated improved clinical outcome scores. Furthermore, the study also included 41 three-level TDR cases and 4 four-level cases, all of which demonstrated similar improvements in satisfaction and VAS pain scores at 24–72 months. These results are promising for the expansion of FDA approval to three levels; however, FDA trials have not yet addressed three-level implantation [5].

## 7. Facet Joint Replacement

While there were early patent applications for facet arthroplasty in the 1980s, the Graf ligament device was one of the earliest attempts at posterior dynamic stabilization and was implanted via the tension band construction of braided polypropylene against titanium pedicle screws of adjacent levels [25]. The goal was to control rotary movement in young patients with axial back pain. Grevitt et al. reported improved ODI scores (59% to 31%) in a retrospective cohort of 50 patients at 2 years [26]. Similarly, Markwalder et al. found improvement in subjective pain perception in (37/41) 90% of patients. However, the design is plagued by resultant lateral canal stenosis, segmental lordosis and early failure in 72% of patients by 2 years, likely due to altered biomechanical strain of the posterior column [27,28]. While this design may be appropriate for patients with spondylolisthesis, it should not be employed in settings of scoliosis or lateral listhesis.

The Total Facet Arthroplasty System (TFAS) (Archus Orthopaedics, Redmond, WA, USA) was designed as an anatomic facet joint arthroplasty system. The design included cement-augmented pedicle screw insertion to anchor the device, which utilizes high-carbon-content cobalt chromium bearings articulating with cobalt chromium spheres to replicate the motion and stability of the facet joints [29] (Palmer et al., 2011). Unfortunately, the system experienced two cases of rod fracture in early clinical trials. The design was ultimately discontinued during phase III clinical trials in light of the hardware failures and following the financial acquisition of Archus in 2009 [30].

Similar to TFAS, however, the Anatomic Facet Replacement System (AFRS) (Facet Solutions Inc., Logan, UT, USA) was an anatomically designed facet prosthesis also utilizing pedicle screw fixation to affix wear-resistant alloy cobalt–chromium molybdenum-articulating surfaces. A finite element analysis (FEA) and parallel in vitro biomechanical study from 2007 demonstrated that the AFRS can predictably restore native facets and intradiscal pressures throughout the spinal range of motion [31]. This datum suggested that the device may be able to restore native biomechanics and therefore restore native adjacent segmental motion, theoretically reducing abnormal contact stress and lowering the risk of adjacent segment disease. Sjovold et al. produced a second FEA and parallel biomechanical study to evaluate the pull-out strength of the implant compared to a rigid posterior fusion construct [32]. The group found that rigid fixation devices were subjected to greater implant loads in extension and lateral bending. They also confirmed a normal intradiscal pressure through the range of motion [32].

The ACADIA device (GLOBUS Medical, Audubon, PA, USA) is a non-anatomic facet joint replacement system available outside the United States. Dryer et al. conducted an IDE and performed a prospective randomized trial with 158 patients randomized to either standard PLIF or the ACADIA trial group [33]. Outcomes demonstrated 52% follow-up at 2 years and found no significant difference between groups with regards to VAS, ZCQ and ODI scores from pre-operation to post-operation at 2 years [33]. The group therefore concluded that the ACADIA implant was a reasonable alternative to PLIF. The ACADIA implant has not been granted FDA approval in the United States but is available for elective intervention outside the United States. Notably, a recent case series of five patients demonstrated a metal-on-metal (MOM)-associated reaction in two (40%) patients necessitating subsequent explanation with PLIF at less than 2 years post-operation [34]. The occurrence of metal-on-metal crevice corrosion is a well-documented danger in orthopedic implant design and may signal a critical design flaw of the ACADIA implant precluding further utilization and FDA approval [35].

**Figure 2:**
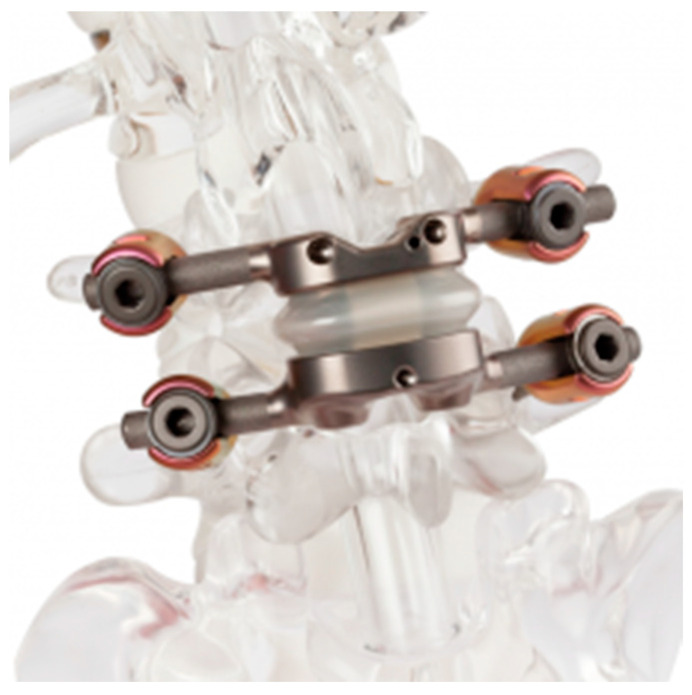
TOPS system (courtesy of FDA: https://www.fda.gov/medical-devices/recently-approved-devices/tops-system-p220002 (accessed on 1 April 2025)).

The Total Posterior Spine (TOPS) system (Premia Spine Ltd., Nowalk, CT, USA) (Figure 2) is a non-anatomic facet arthroplasty system that received FDA approval for use in the United States for patients between 35 and 80 years of age with grade 1 spondylolisthesis of L3–L5 in June, 2023, but has been on the global market since 2012. The device consists of four pedicle screws placed at adjacent levels with metal plates and a central internal motion device surrounded by a polycarbonate boot.

Smorgick et al. reported 11-year post-operative outcomes for 10 patients who underwent isolated total facet arthroplasty with the TOPS system for neurogenic claudication and single-level spondylolisthesis of L4–L5 [36]. The group reported sustained improvements in VAS (5.6 to 1.4), ODI (49.1 to 17) and SF-36 (43.2 to 70.9) at 11 years compared to their pre-operative states. Four (36%) patients experienced adjacent segment disc degeneration by 11 years, without requiring surgery. One early device failure occurred and required revision at 6 weeks. Results were similar in a separate group of ten patients at 5 years of follow-up [37].

A multi-staged prospective randomized trial group has demonstrated promising findings over the past several years [38,39,40]. Patients were randomized to either decompression and fusion or decompression and total facet arthroplasty. A total of 321 patients were enrolled and randomized in a 2:1 fashion with 113 arthroplasty (51.6%) and 47 (46.1%) fusion patients with 2 years of completed follow-up or early failure [40]. The arthroplasty cohort demonstrated improved VAS (back pain), ODI and ZCQ and lower higher rates of adjacent segment disease [40]. There was no significant difference in complications or revision rates (11.5% vs. 10.6%). Of note is that there were no statistical comparisons of the time points in which complications occurred. Furthermore, 6.8% (14/206) of the patients in the arthroplasty group suffered a dural tear compared to only 2.2% (2/93) in the fusion group (*p* = 0.16). Notably, the rate of adjacent segment disease in the arthroplasty group was 0% compared to 5% in the fusion group, demonstrating successful proof of concept despite the concerns surrounding dural tear events [40]. Currently, there is active monitoring and continued data collection with multiple clinical trials ongoing [38,39,40].

Future directions for total facet arthroplasty will need to include considerations of long-term safety; specifically, the TOPS device mechanism will require continued observation. Based on the present data, there may be a paradoxical increase in adjacent segment disease within arthroplasty cohorts compared to fusion; however, larger groups and longer follow-ups will be required. Minimally invasive strategies may also be advantageous for patient recovery. Similarly, patient-specific implantation and device design that respects the native facet joint architecture with thoughtful design criteria could also provide improved patient outcomes.

## 8. Dynamic Stabilization

Posterior dynamic stabilization (PDS), as discussed previously, was first described in the 1980s, with early attempts including the Graf ligament device [25]. The primary goal of PDS is to allow micromotion for better fusion, decrease rigidity and allow improved load sharing, which has been associated with decreased risk of ASDi and ASDe. Broadly, PDS is achieved via 1. altered rod shape, 2. elastic rod materials, 3. polyaxial pedicle screws, 4. semi-dynamic plating devices and 5. unique mechanical constructs, or some combination of the above. There has been a myriad of devices released to achieve PDS; however, there are several examples worthy of discussion, notable for both clinical successes and failures, many of which warrant further investigation.

The Dynamic Neutralization System (Dynesys) was developed in 1994 by Gilles Dubois (Centerpulse Orthopaedics Ltd., Winterthur, Switzerland), with the first results formally published in 2002 [41]. This original device was designed to connect standard pedicle screws with hollow rods made of PCU with PET cords inside the hollow shell, allowing for flexible multidirectional control [41]. Early indications included spinal stenosis and DJD. Importantly, this design differed from the Graf ligament because it theoretically prevented foraminal collapse with spinal extension. The Dynesys received 510 k approval in 2004; however, it has failed to obtain FDA standalone approval for dynamic stabilization, largely due to inconclusive data on its efficacy and long-term outcomes.

A recent literature review outlined the short-term, mid-term and long-term results of the Dynesys device over the past 20 years [42]. Short-term results at 2 years have shown no significant differences in ODI, VAS, range of motion of adjacent segments or risk of adjacent segment disease [42,43]. A more recent meta-analysis of mid-term and long-term time points evaluated 17 studies with 1296 patients which found that the Dynesys patients experienced more natural index and adjacent level motion associated with improved back pain compared to the instrumented fusion cohort’s levels [44]. The longest time point included was 93.6 months, with 58 patients (33 in the Dynesys group), in a study conducted by Bredin et al. [45]. This retrospective comparative cohort study found significantly better VAS (1.8 vs. 3.6), ODI (14.6 vs. 19.4) and decreased rates of adjacent segment disease (12.1% vs. 36%) at final follow-up compared to the rigid instrumented fusion cohort [45].

One prospective randomized controlled trial exists on the product and was completed in China [46]. Results at 2 years demonstrated significantly greater ROM of the operated segment without significant differences in VAS and ODI when compared to the fusion cohort [46]. While the Dynesys device may protect normal spinal motion, large, long-term level I studies will be critical to elucidate the validity of these preliminary findings.

Although there have been some promising data with the Dynesys, other flexible rod systems have demonstrated poor outcomes or a need for further monitoring. The Accuflex Rod System produced by Globus Medical (no longer on the market) was a standard 6.5 mm titanium rod with a variable circumferential helical cut to allow customized flexibility [47]. Mandigo et al. reported results from 170 patients (54 in the Accuflex cohort) who underwent posterior instrumented fusion and found similar fusion rates without a difference in clinical outcomes [47]. However, a report of 20 patients demonstrated fatigue failure at a rate of 22.22%, requiring revision surgery [48]. The Accuflex Rod System might be prone to catastrophic mechanical fracture due to cyclic failure and is no longer offered on the US market and should not be implemented.

The Isobar TTL System (Scient’x) is a titanium rod with a damper component in the longitudinal axis and first received FDA clearance in 1999. The damper provides 2.25 degrees of angular range of motion in flexion–extension and lateral bending without axial rotation restriction [49]. Guan et al. recently published a systematic review of current results for the Isobar system [49]. When evaluating fusion surgery and hybrid fusion surgery, the group found greater than 88.5% fusion at final follow-up [49]. Barrey et al. found a fusion rate of 89% by ten years with stable symptomatic improvement after fusion with the Isobar system [50]. Importantly, there is a paucity of literature evaluating the rate of ASD; however, the small cohort in the study conducted by Barrey et al. does report a low rate of only 44.4% by ten years. Notably, the largest series of patients treated with the Isobar system was published by Perrin et al., with a collection of 800 patients who underwent dynamic stabilization, dynamic fusion and hybrid fusion, with an overall fusion rate of 98% without any mechanical complications [51]. In another cohort series by Li et al., the incidence of adjacent segment disease was 15% (6/40) at 79 months [52]. Unfortunately, this was a retrospective case series, and there was no direct comparison between PDS instrumentation and standard rigid fusion instrumentation. Thus, limited data have been reported on ASD after PDS with flexible rods, particularly Isobar. In short, the Isobar system may be indicated for young patients with single-level pathology, including spinal stenosis and spondylolisthesis, and may be better suited for hybrid, non-fusion techniques. Further high-quality investigations are warranted.

Compared to standard pedicle screws in rigid fixation, dynamic pedicle screws allow varying degrees of freedom and motion between the fixation (threaded) and rod fixation (tulip). Hayati et al. reported on a retrospective cohort series of 101 patients treated with standard pedicle screw fusion or dynamic pedicle screw fixation and found a non-significant decreased rate of radiographic adjacent segment disease at 79 months post-operation in those with dynamic screws [53]. Furthermore, these results did not correlate to any significant clinical benefit, including VAS, ODI or complication profiles. A prospective randomized double-blinded multicenter study by Meyer et al. evaluated standard instrumented fusion with posterior stabilized and pedicle-based dynamic stabilization without fusion [54]. The dynamic group received the Cosmic MIA system, which has a hinged joint between the screw head and the threaded screw, allowing sagittal motion [54]. The group found that non-instrumented dynamic stabilization was non-inferior to standard instrumented fusion, with no significant differences between the groups with regard to VAS and ODI at 24 months. This study may suggest that posterior or posterolateral dynamic fixation without fusion is a sufficient treatment for DJD of the lumbar spine compared to standard instrumented fusion [54].

Classic titanium rods have a high modulus of elasticity (110 GPa), which can lead to immediate segmental stability; however, they may be related to stress shielding and pseudoarthrosis. Polyetheretherketone (PEEK) is a material with a modulus of elasticity of only 3.6 GPa, which allows for a more flexible construct, increased load sharing to the anterior column, improved stress-shielding and, overall, a more natural physiologic loading of the spinal column [55].

Several meta-analyses exist on the topic of PEEK rod implementation for lumbar fusion [56,57]. Li et al.’s was the most recent and evaluated eight prospective and seven retrospective studies comparing rigid titanium rods and PEEK rod stabilization with intervertebral bone grafting at a minimum of 6 months from surgery [57]. Based on the random effects model, Li et al. concluded that the PEEK flexible rod group had superior improvement in VAS, ODI and JOA scores and fusion rates by 12 weeks from surgery sustained to final follow-up. Critically, while Li et al. (2023) included eight prospective studies, the level of evidence was limited by retrospective data inclusion and limited follow-up length. Furthermore, the group was unable to compare the incidence of adjacent segment disease—the primary theoretical benefit of flexible posterior instrumentation. Overall, the evidence for PEEK rod implementation remains low quality and should be improved over time.

The DSS-HPS^®^ System (Paradigm Spine, Germany) is a unique modular system with an internal coupler designed to limit spinal range of motion to 50% of the physiologic motion with an allowed 2 mm of displacement (1.33 mm in tension and 0.66 mm in compression), maintaining a fixed center of rotation [58]. Paradigm Spine implemented an internal registry of patient information for the long-term tracking of clinical outcomes; however, the data remain unpublished and accessible only to Paradigm Spine internal use. Few studies have been published on clinical outcomes; however, Angelini et al. reported results of 27 consecutive patients treated with hybrid stabilization [59]. The group found that 40.7% of the patients experienced disc space degeneration at both the instrumented levels and adjacent levels at one year after surgery [59]. They concluded that the device may not function as well as other systems such as the Dynesys with regards to ASD. The DSS system remains unavailable in the USA.

The landscape of posterior dynamic stabilization is tenuous. While multiple products are currently available for implementation in lumbar spine surgery, all products with FDA approval are approved for only single-level instrumentation in grade 1 spondylolisthesis. Off-label utilization is a common occurrence across many fields in medicine and allows further investigation and understanding of the use criteria; however, it can cause patient harm. Future directions for posterior dynamic stabilization systems include attaining a higher quality of evidence with prospectively designed studies, investigating multi-segment disease and combination treatment with multi-column fixation and arthroplasty, among other focused investigational study strategies. From an engineering perspective, flexible rods present a design challenge in a construct innately at risk for cyclic load failure. With regard to future development, currently, many companies are creating their own version of a PEEK rod or other dynamic rod due to its documented success and hope to gain market share. This competition will ideally lower the cost of implants over time. Future development of technology such as microsensors for stress/strain evaluation in vivo, microprocessor dynamic control and patient-specific implants could provide helpful insight and improve patient outcomes.

## 9. Total Joint Arthroplasty

**Figure 3:**
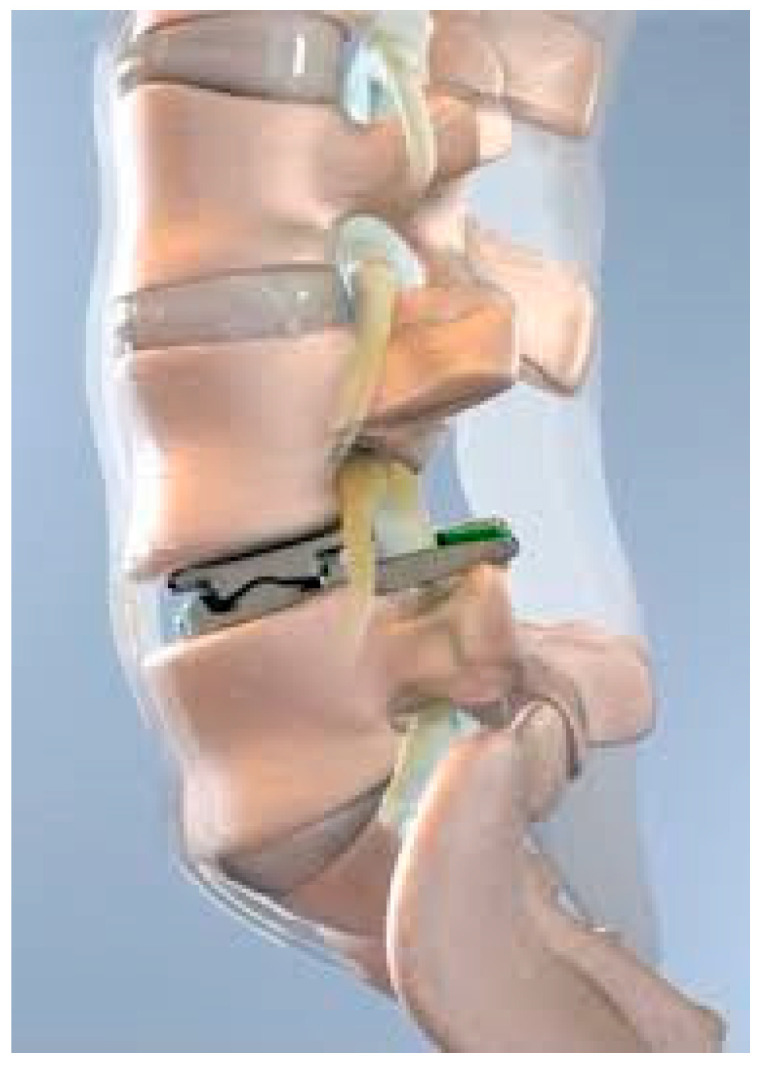
MOTUS 3Spine (courtesy of Nel et al. [62]).

Total joint spinal arthroplasty (TJR) involves the replacement of both the anterior and posterior columns simultaneously from a single approach, including the degenerative intervertebral disc and facet joints, to relieve pain or neural compression while preserving motion and spinal alignment. TJR in theory fills the gap left off by isolated disk replacement as a treatment for patients that have not only disk degeneration but also concomitant facet degeneration as well. Currently, TJR is indicated for grade 1 spondylolisthesis, recurrent disc herniation with severe disc degeneration, severe foraminal or central stenosis requiring extensive facet or pars removal and degenerative disc disease with concurrent facet arthrosis [60]. Contraindications include trauma, tumors, infections, severe deformity and osteoporosis, with pre-operative bone density assessment recommended to ensure adequate implant fixation [60]. The only currently available implant for total spinal arthroplasty in the US is the MOTUS (3Spine, Chattanooga, TN, USA) (Figure 3), which is used at levels L1-L2 to L5-S1. The TJR implant is designed to replace both the intervertebral disc and facet joints while allowing for wide neural decompression. The surgical approach involves a posterior bilateral transforaminal technique, incorporating laminectomy, bilateral facetectomy and partial discectomy to achieve decompression, followed by implantation of the MOTUS device to reconstruct the motion segment.

A key consideration in the long-term success of TJR of the spine is implant durability, particularly wear resistance, which directly impacts device longevity and clinical outcomes. Siskey et al. evaluated the wear performance of a vitamin-E-stabilized highly crosslinked polyethylene (VE-HXLPE) lumbar TJR, designed to replace both the intervertebral disc and facet joints via a posterior approach [61]. Standard wear testing demonstrated a low mean wear rate of 1.2 ± 0.5 mg per million cycles (MC), significantly lower than traditional anterior disc replacement designs, which range from 2.7 mg/MC to 13.8 mg/MC. Abrasive wear testing resulted in a similar wear rate (1.1 ± 0.6 mg/MC), indicating resistance to third-body wear. Impingement testing showed slightly increased wear rates, ranging from 1.7 ± 1.1 mg/MC (smallest implant size) to 3.9 ± 1.1 mg/MC (largest size). Importantly, no mechanical failures were observed, and the VE-HXLPE implant outperformed traditional ultra-high-molecular-weight polyethylene under similar conditions. These findings support the FDA-regulated clinical trial of VE-HXLPE TJR and highlight its potential as a durable, motion-preserving alternative to both fusion and conventional ADRs. The material’s superior wear resistance and stability suggests it may reduce long-term complications such as osteolysis and implant failure, which are common concerns with traditional polyethylene designs.

Polyethylene wear is not the only area where TJR shows promise; early clinical outcomes suggest it may offer comparable or improved symptom relief and functional recovery compared to traditional fusion procedures. A recent study by Sielatycki et al. compared posterior-based lumbar TJR to transforaminal lumbar interbody fusion (TLIF) for degenerative lumbar conditions requiring surgical intervention [60]. The TJR implant was evaluated for its ability to provide motion preservation while allowing for wide neural decompression. The study conducted a retrospective analysis of 208 propensity-matched patients, with 52 undergoing TJR and 156 undergoing TLIF. Patient-reported outcomes at 3 and 12 months post-operation were measured using the Oswestry disability index (ODI) and Numeric Rating Scale (NRS) for back and leg pain. Both groups showed significant improvements at 3 months, but at 1 year, LTJR continued to show improvements in ODI and NRS scores, whereas the TLIF group plateaued. Specifically, the LTJR group had a significantly lower ODI at 12 months (12.4 ± 12.8) compared to TLIF (23.8 ± 17.3, *p* < 0.001), and lower NRS back pain scores (2.1 ± 2.3 vs. 3.4 ± 2.8, *p* = 0.006). Further analysis demonstrated that LTJR patients had 3.3 times greater odds of achieving a minimal clinical symptom state (MSS) (*p* = 0.001), 2.4 times greater odds of achieving a substantial clinical benefit (SCB) (*p* = 0.028) and 4.1 times greater odds of achieving a minimal clinically important difference (MCID) (*p* = 0.006) compared to TLIF patients. Furthermore, a recent case report by Nel et al. provided the first valuable long-term data on TJR of the lumbar spine, a novel motion-preserving alternative to spinal fusion for degenerative lumbar disease [62]. In this case study, the authors presented the first two patients to undergo lumbar TJR, both of whom had severe degenerative lumbar disease with chronic, refractory back and leg pain. Sixteen years post-operation, both patients reported complete and sustained symptom resolution, with full functional recovery and unrestricted participation in daily and occupational activities. Importantly, long-term imaging demonstrated no evidence of adjacent segment degeneration, implant failure, or progressive arthropathy. These findings suggest that lumbar TJR may provide durable pain relief and functional benefits in both the short term and the long term while mitigating the adverse effects of fusion. Furthermore, the lumbar TJR implant has demonstrated favorable wear properties and the potential for long-term durability. While further research and long-term trials are needed, these findings support the continued exploration of TJR as a viable option for select patients with lumbar spine degeneration.

## 10. Discussion

The gold standard for surgical management of lumbar spine degeneration has been spinal fusion, despite the known complications and associated risk of additional surgery. Lumbar arthroplasty is a motion-preserving alternative to spinal fusion for surgical treatment of lumbar degeneration. (Table 1) Arthroplasty has not been widely accepted due to concerns regarding implant survival, revision risk and a lack of long-term follow-up data. The lack of long-term data has often been cited as the reason for apprehension toward lumbar arthroplasty. The three-joint complex of a single lumbar spine segment adds complexity to the prospect of lumbar arthroplasty. Therefore, patient selection and selection of surgical treatment are critical for clinical success. Total disk replacement for isolated disk degeneration has led the way in the field, with recent promising results in 21-year follow-up data from a large cohort [11]. With these recent favorable results from the long-term experience of Thierry and Guyer, the durability and long-term efficacy of lumbar arthroplasty has been further validated as a favorable and reliable alternative to fusion in patients without facet arthropathy [11,23]. Furthermore, success in lumbar disk arthroplasty at one and two levels has demonstrated that TDR is a reasonable multilevel treatment as well. Indications for lumbar arthroplasty have been generally limited to early disk degeneration; as the field progresses, the clinical pathway for arthroplasty will need to be critically evaluated as indications expand. Pitfalls such as the misdiagnosis of spinal epidural lipomatosis upon MRI could lead to inappropriate surgery and should be considered during patient evaluation [63]. Also, as mentioned previously, concomitant facet arthropathy should be identified as a contraindication to isolated disk arthroplasty. 

Addressing lumbar facet pathology has been a challenging hurdle to overcome to expand the indication of lumbar arthroplasty. While devices such as TOPS have shown some early success, other implants, such as some dynamic rods, have demonstrated notable clinical failures, highlighting the need for further observation, monitoring and implant development [40,48]. Total joint arthroplasty (TJR), which addresses the degenerative disk as well as the facets from the same posterior approach, has shown good results in both short- and long-term studies. These are exciting advances for motion-preserving treatment of posterior column degeneration. Although limited, current data point to the possible expansion of lumbar arthroplasty as an option for lumbar degeneration beyond isolated disk disease. While lumbar fusion will likely remain the workhorse salvage procedure for advanced lumbar degeneration, the progress in lumbar arthroplasty is a promising alternative for early degeneration across all three joints of a lumbar spinal level. When coupled with advances in navigated technology and minimally invasive techniques, arthroplasty has the potential to provide customized treatment options to address the challenge of lumbar degeneration. In totality, these findings indicate a cautious optimism toward the future of lumbar spine arthroplasty and motion-preserving surgery.

## 11. Conclusions

Lumbar arthroplasty has the theoretical potential to provide a motion-preserving alternative to lumbar fusion for patients with lumbar spine degeneration. Although substantial long-term outcome studies are still needed, there have been promising data thus far indicating a cautious optimism toward the future of motion preservation in degenerative lumbar spine surgery.

## Figures and Tables

**Table 1:** Summary table comparing advantages and disadvantages of different spinal implants.

Implant Type	Advantages	Disadvantages
Lumbar Interbody Fusion (LIF)	- Widely practiced and understood surgery with multiple approaches and techniques to address the degenerative lumbar spine and deformity correction - Current standard of care - Minimally invasive surgery is an option - Able to extend to multiple levels (useful in complex or revision settings) - Acceptable short-term clinical outcomes	- Pseudoarthrosis - Hardware failure - Loss of motion at the fused level accelerates adjacent segment degeneration
Artificial Lumbar Disc Arthroplasty	- Leading alternative construct in preserving motion at the index lumbar level - Low adjacent segment stress forces with lower rates of adjacent segment degeneration - Lower levels of early disability scores, higher patient satisfaction and shorter length of hospital stay compared to LIF	- Only addresses degenerative intervertebral disc; unable to address posterior column pathology - Contraindicated in facet degeneration, poor bone quality and significant deformity - Anterior approach risks (vascular/ureter injury) - Concerns with implant stability and durability may increase risk of revision surgery - Currently only two FDA-approved implants that can be used for one to two contiguous levels
Facet Joint Replacement	- Addresses posterior column - Offers patient-specific instrumentation and device design that respects the native facet joint architecture - Restores native biomechanics while preserving motion - Minimally invasive surgery is an option	- Limited FDA-approved options due to mixed results and high complication rates among different implant options - Latest devices are limited to single-level spondylolisthesis of L3–L5 - Some early evidence of paradoxical increases in adjacent segment disease, although larger long-term studies are needed
Flexible Rods	- Allow micromotion, decrease rigidity and improve load sharing with a theoretical decreased risk of adjacent segment disease - Multiple implant options for posterior dynamic stabilization	- Despite multiple options, the data are largely inconclusive on the efficacy and long-term outcomes due to low-level evidence - All FDA-approved products are only approved for single-level instrumentation in grade 1 spondylolisthesis - Off-label use is common - Implant design and construct is innately at risk for cyclic load failure
Total Joint Arthroplasty (TJR)	- Addresses both anterior column and posterior column pathology while preserving motion and spinal alignment - Single posterior approach utilizing bilateral transforaminal technique to replace both the intervertebral disc and facet joints, providing wide neural decompression - Improved implant durability and long-term success secondary to superior wear resistance rates from using vitamin-E-stabilized highly crosslinked polyethylene (VE-HXLPE)	- Currently only one implant available for implementation - Limited use at L1–L2 and L5-S1, highlighting the importance of appropriate patient selection - Current data are promising but remain limited due to low-level evidence

## Abbreviations

The following abbreviations are used in this manuscript:

TDR	total disk replacement
TJR	total joint replacement
ASDe	adjacent segment degeneration
ASDi	adjacent segment disease
